# Supplementary material for: New Insight into the Function of Dopamine (DA) during Cd Stress in Duckweed (Lemna turionifera 5511)
Source: Plants (Basel). 2023 May 16;12(10):1996. doi: 10.3390/plants12101996 (PMC10221877; doi:10.3390/plants12101996)
Supplement: Supplementary file 1 [file plants-12-01996-s001.zip › plants-2166195-supplementary.pdf]

Table Supplements:

Table S1. Gene expression in case of "DA vs CK" in photosynthesis and antenna proteins

**Description**	**GeneID**	**DA_readcount**	**CK_readcount**	**log2FoldChange**	**pval**	**padj**
Photosystem II 10 kDa polypeptide (PsbR)	Cluster-4169.8284	62360.62122	100815.4174	-0.69301	4.88E-06	9.57E-05
Photosystem II 22 kDa protein 1 (PsbS)	Cluster-4169.1008	1.911620616	1.350504431	0.51275	0.7744	0.94821
Photosystem II 22 kDa protein (PsbS)	Cluster-4169.8589	54613.63288	67280.84829	-0.30094	0.019956	0.1053
Photosystem I reaction center subunit IV (PsaE)	Cluster-4169.6111	16749.43962	19458.78344	-0.21632	0.10093	0.34378
Photosystem I reaction centre subunit IV (PsaE)	Cluster-4169.9246	52.38926601	38.94959594	0.42608	0.2721	0.63323
Photosystem I subunit O (PsaO)	Cluster-4169.11063	35632.89131	53691.85312	-0.5915	0.00071	0.007045
light-harvesting complex I chlorophyll a/b binding protein 3 (LHCA3)	Cluster-4169.8951	50379.14067	48769.02663	0.046859	0.81307	0.94821
light-harvesting complex II chlorophyll a/b binding protein 1 (LHCB1)	Cluster-4169.13387	1292.110178	913.7879608	0.49969	0.077927	0.28846
light-harvesting complex II chlorophyll a/b binding protein 1 (LHCB1)	Cluster-4169.7570	96400.33153	87890.26868	0.13333	0.64389	0.90228
light-harvesting complex II chlorophyll a/b binding protein 1 (LHCB1)	Cluster-4169.9400	26702.82844	50947.03216	-0.93201	0.000538	0.005582
light-harvesting complex II chlorophyll a/b binding protein 1 (LHCB1)	Cluster-37.0	1.27819834	1.666293631	-0.37636	0.84134	0.95212
light-harvesting complex II chlorophyll a/b binding protein 1 (LHCB1)	Cluster-4169.4567	1281.946821	651.2122448	0.97696	0.000622	0.006324
light-harvesting complex II chlorophyll a/b binding protein 1 (LHCB1)	Cluster-4169.8577	276730.62	333455.5685	-0.26901	0.28997	0.65551
light-harvesting complex II chlorophyll a/b binding protein 6 (LHCB6)	Cluster-4169.3893	18860.58903	14702.41304	0.35931	0.12797	0.40293

Table S2. Gene expression in Cd accumulation and detoxification proteins

Description	GeneID	DA_Cd_readcount	Cd_readcount	log2FoldChange	pval	padj
Vacuolar iron transporter homolog 1	Cluster-842.295	17.87	0.63	4.83	0.000203	0.010926
ribulose-bisphosphate carboxylase small chain	Cluster-842.10020	264.90	30.97	3.10	9.62E-15	1.27E-11
glutathione S-transferase	Cluster-842.9840	5957.78	2273.15	1.39	2.95E-10	1.57E-07
glutathione S-transferase	Cluster-842.8870	2458.37	985.25	1.32	4.00E-06	0.000499
glutathione S-transferase	Cluster-842.1037	1485.06	633.39	1.23	7.73E-12	7.21E-09
glutathione S-transferase	Cluster-842.7825	1606.64	777.67	1.05	9.95E-09	3.12E-06
Multidrug Resistance Protein	Cluster-842.4292	908.58	314.08	1.53	3.76E-05	0.0029845
Nitrate transporter1	Cluster-842.5874	2837.61	1145.46	1.31	4.02E-10	2.04E-07

Alcohol dehydrogenase-like	Cluster-842.11136	420.84	201.53	1.06	0.000244	0.01229
Copper transporter 1	Cluster-842.5588	174.74	71.40	1.29	1.65E-06	0.000243
Succinate dehydrogenase subunit 8A	Cluster-842.9419	772.86	384.52	1.01	8.41E-05	0.005513
fructose-bisphosphate aldolase	Cluster-842.15498	676.29	394.13	0.78	0.000682	0.026116
Fumarate reductase flavoprotein C-term	Cluster-842.8107	4324.27	2704.84	0.68	0.000359	0.016711
starch synthase II	Cluster-842.12068	1375.75	879.62	0.65	0.000213	0.011273
glucosephosphate isomerase	Cluster-842.11743	1056.69	703.30	0.59	0.001399	0.043366
NADP-dependent glyceraldehyde-3-phosphate dehydrogenase-like	Cluster-842.10532	5484.40	3955.89	0.47	0.000154	0.008681
isocitrate dehydrogenase 3 (NAD+)	Cluster-842.9388	5963.89	4362.64	0.45	0.0002	0.01087
fructose-1,6-bisphosphatase	Cluster-842.7980	3883.21	2864.05	0.44	0.000628	0.024461
trehalose 6-phosphate synthase	Cluster-842.9362	942.23	1329.39	-0.50	0.000634	0.024653
trehalose 6-phosphate phosphatase	Cluster-842.10026	165.72	307.12	-0.89	3.42E-05	0.002819
Phosphoenolpyruvate carboxykinase [ATP]	Cluster-842.10423	399.63	854.05	-1.10	2.42E-05	0.002186

Table S3. Gene expression in case of "Cd vs CK" in the hypothesis map of DA signal in plants

Description	GeneID	Cd_readcount	CK_readcount	log2FoldChange	pval	padj
D1 dopamine receptor-interacting protein	Cluster-7365.31904	146.86	24.02	2.60	2.17E-22	6.46E-21
D1 dopamine receptor-interacting protein	Cluster-7365.63336	27.59	95.63	-1.79	0.009543	0.046644
calcium-dependent protein kinase sk5	Cluster-7365.32927	3398.21	1054.15	1.69	4.60E-27	1.64E-25
calcium-dependent protein kinase	Cluster-7365.82136	108.60	10.06	3.43	8.90E-05	0.0006993
calcium-dependent protein kinase	Cluster-7365.83453	226.72	31.05	2.88	1.14E-05	0.00010308
calcium-dependent protein kinase	Cluster-7365.83454	2549.57	603.02	2.08	2.30E-23	7.08E-22
calcium-dependent protein kinase 16	Cluster-7365.83455	33.75	12.04	1.47	0.00019392	0.0014325
non-specific phospholipase c1-like	Cluster-7365.61191	241.16	89.71	1.43	0.00098623	0.0062959
non-specific phospholipase c1	Cluster-7365.70319	40.60	0.00	7.68	0.00020705	0.00152
non-specific phospholipase	Cluster-	386.48	26.98	3.84	4.02E-06	3.87E-05

c4	7365.88318					
non-specific phospholipase c4	Cluster-7365.88317	592.46	166.51	1.83	3.87E-16	8.56E-15
non-specific phospholipase c6	Cluster-7365.100617	360.33	48.90	2.88	5.08E-16	1.12E-14
phospholipase c	Cluster-7365.44357	173.87	42.82	2.03	6.19E-05	0.00049971
phospholipase c	Cluster-7365.44354	3599.17	355.80	3.34	7.55E-91	1.24E-88
non-specific phospholipase c1	Cluster-7365.50892	92.47	0.00	8.87	9.35E-06	8.55E-05
non-specific phospholipase c1	Cluster-7365.50893	1116.73	540.58	1.05	0.0022608	0.013257
protein kinase c terminal domain	Cluster-7365.57240	115.61	41.85	1.47	9.28E-05	0.00072664
protein kinase c terminal domain	Cluster-7365.42998	5461.40	2137.68	1.35	7.23E-16	1.58E-14
calcineurin b-like protein 3	Cluster-7365.58129	709.36	288.74	1.29	8.96E-42	5.10E-40
cyclic nucleotide-gated ion channel 2	Cluster-7365.55503	0.00	167.51	-10.02	4.18E-16	9.23E-15
cyclic nucleotide-gated ion channel 2-like	Cluster-7365.57026	1.54	31.04	-4.34	0.0004331	0.0029885
cyclic nucleotide-gated ion channel 2-like	Cluster-7365.64466	0.00	8.69	-5.75	0.00046292	0.0031755
cyclic nucleotide-gated ion channel 2	Cluster-7365.58747	1465.76	3735.91	-1.35	0.00029622	0.0021097
ki-ras-induced actin-interacting protein-ip3r-interacting domain	Cluster-7365.9445	1486.80	4.55	8.41	1.24E-64	1.21E-62
ki-ras-induced actin-interacting protein-ip3r-interacting domain	Cluster-7365.98293	118.74	0.00	9.23	1.37E-15	2.95E-14
ki-ras-induced actin-interacting protein-ip3r-interacting domain	Cluster-7365.19072	105.42	48.86	1.11	0.0030202	0.017124
ki-ras-induced actin-interacting protein-ip3r-interacting domain	Cluster-7365.87243	75.37	25.67	1.55	8.00E-11	1.23E-09
ki-ras-induced actin-interacting protein-ip3r-interacting domain	Cluster-7365.83154	38.67	7.52	2.38	0.0027923	0.015981
ki-ras-induced actin-interacting protein-ip3r-interacting domain	Cluster-7365.23636	30.71	13.10	1.23	0.0081313	0.040583
ki-ras-induced actin-interacting protein-ip3r-interacting domain	Cluster-7365.23634	76.12	23.27	1.72	0.00054493	0.0036862
ki-ras-induced actin-interacting protein-ip3r-interacting domain	Cluster-7365.23638	57.20	18.85	1.59	0.0017977	0.010798
nitric oxide synthase,	Cluster-	858.82	455.44	0.92	8.11E-12	1.35E-10

oxygenase domain	7365.19921					
camp-dependent protein kinase complex	Cluster-7365.81982	9.76	48.74	-2.31	8.09E-07	8.48E-06
mitogen-activated protein kinase 16	Cluster-7365.30318	168.39	2.41	6.13	1.58E-05	0.00013929
mitogen-activated protein kinase 5	Cluster-7365.62729	3080.16	340.20	3.18	2.16E-38	1.12E-36
mitogen-activated protein kinase 5	Cluster-7365.62727	70.28	13.13	2.43	3.12E-10	4.59E-09
mitogen-activated protein kinase 10-like	Cluster-7365.30708	52.74	120.16	-1.19	0.0099225	0.048255
mitogen-activated protein kinase 8-like	Cluster-7365.30712	14.57	55.74	-1.93	0.0053222	0.028196
mitogen-activated protein kinase 2	Cluster-7365.80797	690.40	258.45	1.42	1.26E-17	3.03E-16
calmodulin	Cluster-7365.66408	1886.85	516.72	1.87	6.17E-16	1.35E-14
calmodulin	Cluster-7365.11509	36.62	14.31	1.35	0.00060462	0.0040506
calmodulin	Cluster-7365.96103	1022.98	14.32	6.17	7.55E-156	3.23E-153
calmodulin	Cluster-7365.55570	8310.66	2363.55	1.81	2.00E-40	1.10E-38
calmodulin	Cluster-7365.17727	2926.29	215.53	3.77	2.14E-33	9.40E-32
calmodulin	Cluster-7365.56939	3283.27	1073.51	1.61	5.84E-46	3.70E-44
calmodulin	Cluster-7365.56941	24.96	7.87	1.65	0.00062065	0.0041457
calmodulin	Cluster-7365.56943	15041.20	4457.76	1.75	1.64E-63	1.58E-61

Table S4. Gene expression in case of “DA-Cd vs Cd” in Ca^{2+} transporter

Description	GeneID	DA_Cd_readcount	Cd_readcount	log2FoldChange	pval	padj
Cyclic nucleotide-gated ion channel 2	Cluster-842.334	233.60	58.37	2.00	2.29E-08	6.51E-06
Adenylate cyclase	Cluster-842.7281	8317.67	5379.79	0.63	6.57E-05	0.004482
Adenylate cyclase	Cluster-842.9111	442.06	298.13	0.57	0.000699	0.026617
Calcium-transporting ATPase 8	Cluster-842.10474	5579.98	8679.19	-0.64	4.19E-05	0.003219

Table S5. Gene expression in DA biosynthesis

Description	GeneID	CK readcount	Cd readcount	log2FoldChange	pval	padj
3-deoxy-D-arabino-heptulosonate-7-phosphate synthase	Cluster-7365.57044	3210.16	9456.94	1.56	5.25E-27	1.87E-25

3-dehydroquinate synthase	Cluster-7365.67842	21.26	116.79	2.46	0.0097021	0.047325
shikimate kinase	Cluster-7365.46020	177.10	39.32	-2.17	1.97E-06	1.97E-05
shikimate kinase	Cluster-7365.56277	51.43	12.29	-2.07	0.00065085	0.0043328
shikimate kinase	Cluster-7365.56278	56.48	6.38	-3.13	1.16E-06	1.20E-05
shikimate kinase	Cluster-7365.61332	103.34	35.50	-1.54	0.002416	0.014053
shikimate kinase	Cluster-7365.61335	601.91	109.18	-2.46	3.83E-68	4.09E-66
shikimate kinase	Cluster-7365.61337	2058.66	274.49	-2.91	6.89E-62	6.35E-60
chorismate mutase	Cluster-7365.39059	54.46	24.68	-1.15	0.00023549	0.0017118
chorismate mutase	Cluster-7365.41153	183.92	74.67	-1.30	0.0049113	0.026282
chorismate mutase	Cluster-7365.46202	22.47	0.00	-7.13	2.81E-09	3.82E-08
chorismate mutase	Cluster-7365.48442	40.58	5.32	-2.92	0.0024548	0.014253
chorismate mutase	Cluster-7365.49125	83.01	0.00	-9.01	5.67E-15	1.16E-13
chorismate mutase	Cluster-7365.61093	32.63	13.38	-1.29	0.010104	0.048989
chorismate mutase	Cluster-7365.65774	602.10	253.98	-1.25	8.29E-13	1.47E-11
prephenate dehydratase	Cluster-7365.102271	62.22	28.78	-1.12	0.00039018	0.0027145
prephenate dehydratase	Cluster-7365.102835	52.09	204.47	1.98	1.01E-14	2.05E-13
prephenate dehydratase	Cluster-7365.27487	226.44	72.89	-1.64	1.88E-08	2.36E-07
prephenate dehydratase	Cluster-7365.39423	985.33	2333.10	1.24	8.21E-14	1.56E-12
aspartate aminotransferase	Cluster-7365.18944	155.79	573.40	1.88	1.72E-06	1.73E-05
aspartate aminotransferase	Cluster-7365.18953	143.68	578.13	2.01	9.33E-24	2.91E-22
aspartate aminotransferase	Cluster-7365.39096	809.02	391.15	-1.05	7.03E-29	2.65E-27
aspartate aminotransferase	Cluster-7365.39102	47.07	15.90	-1.56	8.05E-06	7.43E-05
aspartate aminotransferase	Cluster-7365.39109	80.40	38.37	-1.07	0.001254	0.0078039
aspartate aminotransferase	Cluster-7365.39111	131.74	57.71	-1.19	2.31E-07	2.58E-06
aspartate aminotransferase	Cluster-7365.62079	16.26	102.47	2.65	0.00090284	0.005821
aspartate aminotransferase	Cluster-7365.65048	1479.86	11772.77	2.99	1.33E-252	2.04E-249

aspartate aminotransferase	Cluster-7365.65049	11.55	100.69	3.13	3.41E-21	9.61E-20
aspartate aminotransferase	Cluster-7365.65050	0.27	9.91	4.92	9.70E-05	0.00075621
aspartate aminotransferase	Cluster-7365.70238	0.00	6.42	5.02	0.0040243	0.02209
tyrosine aminotransferase	Cluster-7365.47461	163.18	33.04	-2.30	0.0010137	0.0064527
tyrosine aminotransferase	Cluster-7365.63144	81.13	0.24	-8.26	0.001452	0.0089066
tyrosine aminotransferase	Cluster-7365.96189	234.57	651.15	1.48	2.58E-14	5.04E-13
polyphenol oxidase	Cluster-7365.21550	56.64	19657.11	8.44	7.25E-51	5.18E-49
polyphenol oxidase	Cluster-7365.48622	7753.29	1721.89	-2.17	2.97E-27	1.07E-25
tyrosine decarboxylase	Cluster-7365.39207	54.81	3.95	-3.80	2.36E-07	2.63E-06
tyrosine decarboxylase	Cluster-7365.70755	265.28	75.79	-1.81	2.64E-08	3.27E-07
tyrosine decarboxylase	Cluster-7365.70756	397.66	115.75	-1.78	2.59E-18	6.42E-17
tyrosine decarboxylase	Cluster-7365.98292	1.10	2402.46	11.12	5.57E-36	2.66E-34
tyrosine decarboxylase/	Cluster-7365.98620	2.96	1803.94	9.26	2.43E-40	1.33E-38
tyrosine decarboxylase	Cluster-7365.98862	1.76	639.63	8.58	1.36E-19	3.58E-18
tyrosine decarboxylase	Cluster-10682.0	1.77	38.95	4.53	2.40E-06	2.38E-05
tyrosine decarboxylase	Cluster-10682.1	2.68	18.75	2.76	0.0077702	0.039039

Figure Supplements:

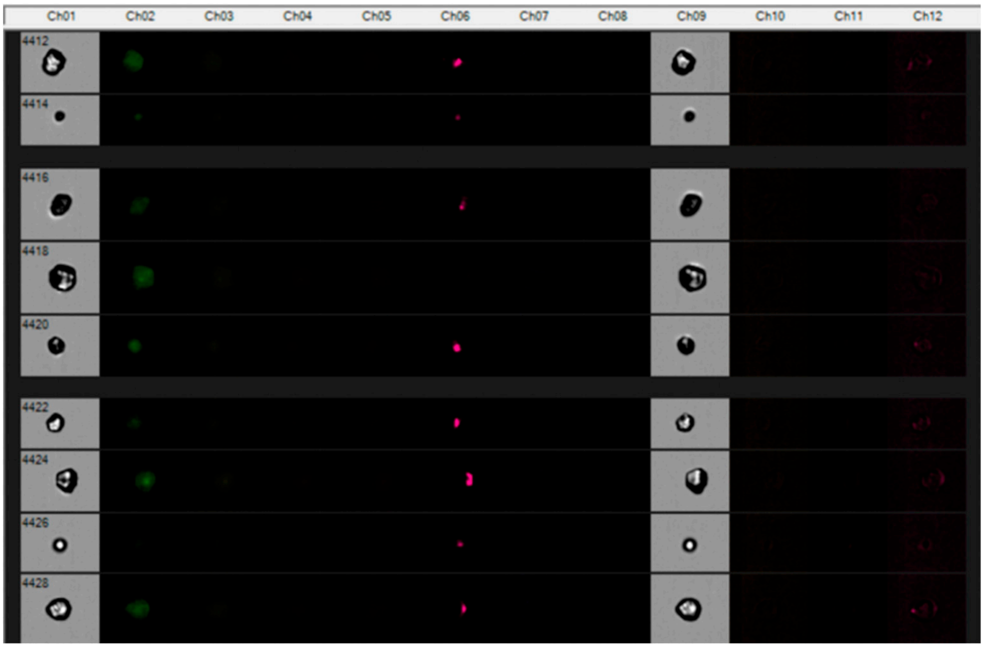

Figure S1: All 12 channels in flow cytometry. Ch01, bright sight; Ch02, the Leadamium signal intensity; Ch05, the chlorophyll autofluorescence.

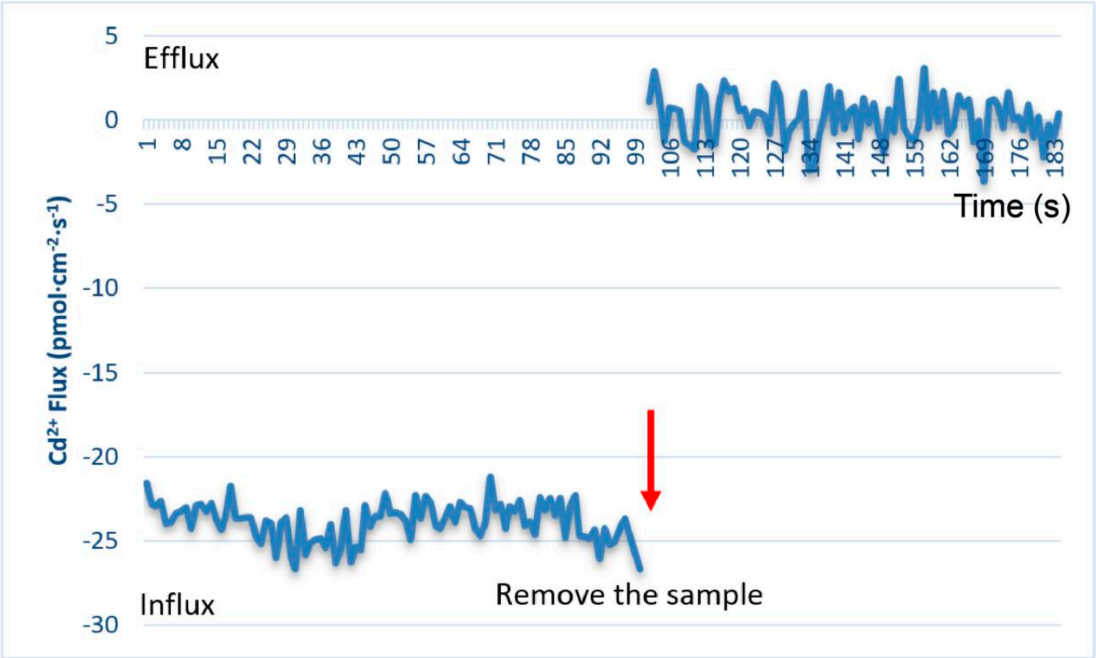

Figure S2: Tested by NMT, Cd flux with or without plant sample. With sample existence, Cd^{2+} flux is -24.14±2.60 pmol cm^{-1} s^{-1}, but after the sample was removed, the flux became very weak and fluctuated at -0.27±3.3 pmol cm^{-1} s^{-1}.

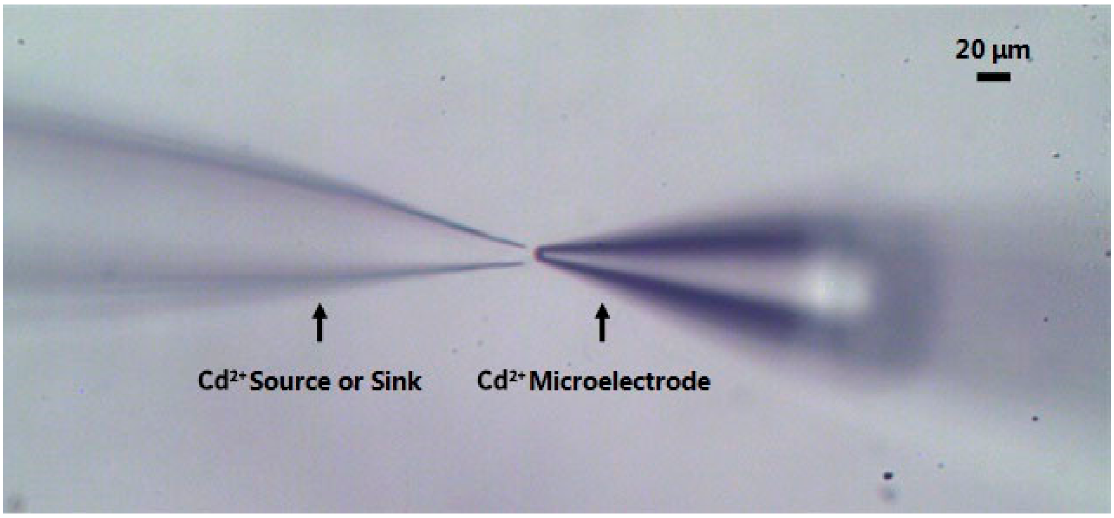

Figure S3： Artificial Cd^{2+} absorbing source was tested by NMT.

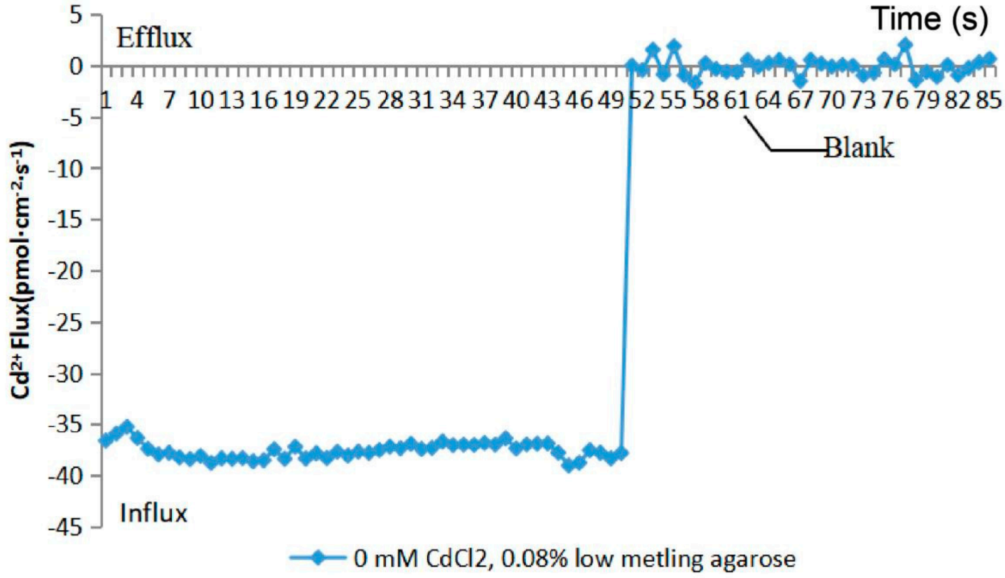

Figure S4: Cd flux of artificial Cd^{2+} absorbing source and blank. An artificial Cd^{2+} absorbing source, with known Cd^{2+} flux direction was adopted to simulate the sample, and NMT was used for detection. Cd flux of source was -37.14±1.89 pmol cm^{-1} s^{-1}, and after remove the source, the background was also shown with flux of 0.17±1.80 pmol cm^{-1} s^{-1}.
